# Extension of the Side Distance Measurement Aspect Ratio in the Measurement of a Slot or Bore Using a Commercial Laser Triangulation Sensor

**DOI:** 10.3390/s24237734

**Published:** 2024-12-03

**Authors:** Jan Hošek

**Affiliations:** Faculty of Mechanical Engineering, Czech Technical University in Prague, Technická 4, 166 07 Praha, Czech Republic; jan.hosek@fs.cvut.cz; Tel.: +420-2-2435-3234

**Keywords:** laser triangulation, bore, slot, distance measurement, aspect ratio, mirror, edge

## Abstract

We propose a new commercial laser triangulation sensor modification to enable the measurement of slots or bores side distance. The study showed the possibility of extending the sensor depth range for a slot or bore side distance measurement using a bypass of the illumination laser beam compared to a simple single mirror attachment to the sensor probe. We derived relations allowing for evaluation of the modified sensor side measurement range in desired depth based on the sensor parameters and the reflective mirror size and position. We demonstrated the functionality of the proposed measurement arrangement with an attachment to the commercial laser triangulation sensor and assessed the side-wall distance measurement. The results show the correct measurement depth and range prediction and the ability to perform side surface distance measurements at depths of more than 3.5 times the slot size.

## 1. Introduction

The rapid development of the manufacturing industry towards high performance, digitization, and intelligently controlled production raises high demands on fast and precise measurement technology as a tool for position and dimensional characterization of various shapes of produced parts. Many types of high-precision displacement sensors exist. Unlike traditional contact probe measuring methods, non-contact sensors are increasingly used for their high precision and contact-free measurement possibilities.

One suitable, well-adopted, non-contact measurement method represents the laser triangulation sensor [1,2,3]. This is because the laser triangulation system can reach micron measurement levels, measure simultaneously, and is cost-effective compared to other measuring principles. However, this sensor can be disturbed by different measuring materials [4,5,6], surface inclines [7,8,9], pointing errors [10,11,12], and edge discontinuities [13]. The measurement issue on high slopes and edges of the surfaces makes measuring the inner surfaces of parts difficult.

It led to the development of various other optical inner diameter and/or profile measurement methods using a circular ring [14,15] and structured or diffractive light projection [16,17]. Systems using the laser triangulation method were also developed, but with a special design to fit the bore size to perform the measurement [18,19,20,21]. It limits the bore diameter that can be measured to the size of inserts provided with laser distance sensors. If the bore diameter is smaller than the sensor size, the system must be provided with a mirror attachment to allow for the measurement inside the bore [22,23,24]. Recently, we analyzed the maximum measurement depth for side wall distance measurement, in general, using a commercial laser triangulation sensor and a single mirror placed on the sensor attachment [25]. We found two extreme configurations: a parallel sensor arrangement (PSA) and a symmetric sensor arrangement (SSA). We focused on the SSA arrangement and found that the accessible depth is always limited by the sensor’s laser illumination beam obscuration by the top edge of the measured surface. Our motivation for this paper is to derive relations suitable for the design of a particular commercial triangulation sensor modification with an attachment to allow for the side distance measurement in a slot with a high aspect ratio without reducing the sensor measurement accuracy guaranteed by the sensor producer. We focused on the PSA arrangement-based attachment design and derived relations for the attachment system geometry based on the desired side distance measuring range in the measuring depth. We also proposed extending the sensor’s accessible depth beyond the limits of PSA and SSA arrangements using a bypass for the laser illumination beam. It allows for increased slot or bore measurement aspect ratio by reaching the measurement depth limited by the sensor’s maximum working range.

This work introduces the general principle of a laser triangulation sensor in Section 2. The theoretical relations describing the laser spot reflections in the proposed sensor arrangement using the mirror attachment are derived for the principle ray in Section 3. It also allows for evaluation of the maximum accessible measurement depth and side distance measurement working range. The probe designed for experimental verification is described in Section 4. Section 5 proves the system functionality using vertical surface distance measurement demonstrated with a sample block. The results are focused on the limits of the sensor linearity and scanning range caused by the laser spot imaging beam obscuration by the measured block edge. Section 6 summarized all the important findings of the article.

## 2. Principle of Laser Triangulation Sensor

**Figure 1:**
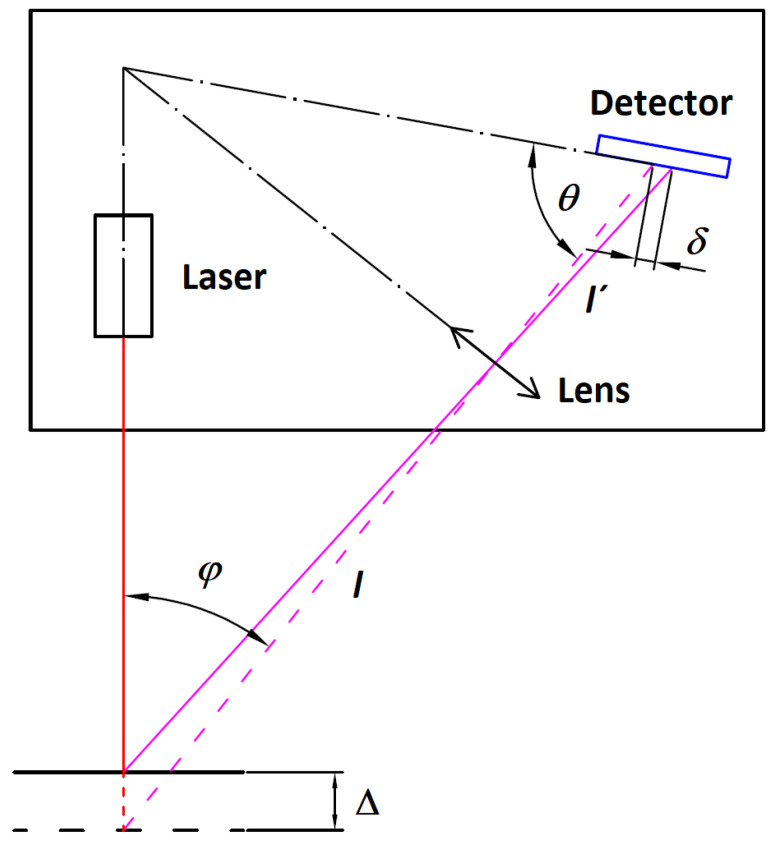
Principle of Laser Triangulation Sensor. The red line represents the focused illumination laser beam. Magenta lines represent laser spot imaging beams through the lens to the sensor’s detector. The detector inclination meets Scheimpflug’s condition.

The general principle of a laser triangulation sensor is shown in Figure 1.

Laser triangulation probe distance measurement uses a focused laser beam projected toward the measured object’s surface, where a laser spot is formed. The actual laser spot position indicates the position of the object’s surface during its motion. The light scattered from the laser spot propagates back to the sensor’s imaging lens. The sensor’s lens field of view covers the whole sensor working range and forms an image of the laser spot in the plane of the linear photodetector. To observe the movement of the object surface along the illumination laser beam, there must be a non-zero angle *φ* between the laser beam and the sensor’s imaging optics axis. The Scheimpflug’s condition [26,27]:

(1) $$ltan\varphi =l^{\prime }tan\theta$$

is applied to the sensor’s design, allowing imaging between conjugated planes when the lens’s optical axis is inclined toward the object or the image plane.

The geometrical relation between the laser spot position shifted due to the object displacement Δ, and the image displacement *δ*, can be expressed with the triangle similarity principle:

(2) $$\delta =\frac{\Delta l^{\prime }sin\theta }{lsin\theta \mp \Delta sin\left(\varphi +\theta \right)},$$

where “+” in the formula is applied if the surface moves up from the reference position, otherwise, is it “−”. Distance position data are evaluated with the laser spot image center extraction algorithm [28,29,30]. A detailed optimization technique for the laser triangulation sensor design can be found in [31,32].

## 3. Analysis of the Laser Triangulation Sensor with a Mirror Attachment

Application of the mirror or prism reflections within the laser triangulation sensor design was successfully used for the solution of various issues related to the data acquisition accuracy of the sensor, such as the measurement environment, the surface properties, or other inherent properties of the sensor [33,34,35,36] or miniaturization of the sensor system [37,38]. We aim to use a mirror reflection to redirect the sensor’s working space from the original direction towards the vertical surface S. The general description of the mirror application for distance measurement of a vertical surface is shown in Figure 1.

An inclination of the laser triangulation sensor under an angle *α* is a standard way for vertical surface distance measurement. It allows placement of the measured surface within the sensor’s working range, but the surface slope strongly affects the sensor’s distance value. Tree conditions assuring the sensor’s distance processing within the sensor producer uncertainty limit determine a necessary mirror position.

(a) The laser illumination beam must be incident perpendicular to the measured surface.
(b) The mirror reflection surface has to be symmetrically placed between the original laser beam direction and the reflected laser beam direction.
(c) The minimum and maximum distance of the measured surface S from the mirror tip must fit the sensor’s working range under mirror reflection.

The selection of a particular triangulation sensor intended for modification to be able to measure side distances provides the design’s first input parameter *A*, the sensor’s imaging lens aperture LA distance from the illumination laser beam. Using this *A* value, we can evaluate the corresponding angle *φ* between the laser illumination beam and the laser spot imaging beam for any distance *B* within the sensor working range from the distance *d_min_* to *d_max_* given in the sensor datasheet, using the relation:

(3) $$tan\varphi =\frac{A}{B},$$

It allows us to evaluate the φ angle range for all sensor working distances.

**Figure 2:**
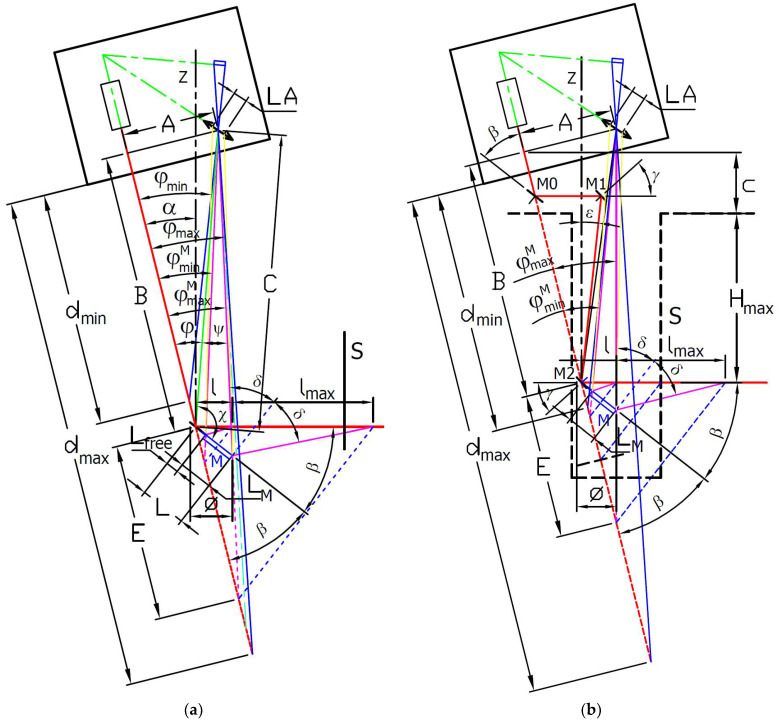
(**a**) General configuration of a commercial laser triangulation sensor with a mirror allowing for the distance measurement of a vertical surface S; (**b**) Configuration of a commercial laser triangulation sensor under condition *α* = *φ^M^_max_* with a bypass of the illumination beam using mirrors M0-M2 allowing for extension of the accessible depth of distance measurement of a vertical surface S.

According to Figure 2a, *B* is the distance where the reflection mirror M of the designed attachment crosses the illumination laser beam to change the direction of the laser beam towards the side wall of the measured surface S. The value of distance *B* can be selected as the design input parameter in the range from *B_min_* to *B_max_* given by the minimum and maximum sensor working distances. It will be shown that it is more convenient to evaluate the *B* distance from the other input parameters.

The distance *C* from the sensor lens aperture to the reflection mirror M position is given by the following relation:

(4) $$C=\frac{A}{sin\varphi }.$$

Under consideration of perpendicular illumination of a vertical surface S, the aimed direction reflected laser illumination beam is horizontal and perpendicular to the vertical axis *z* of the whole sensor setup from which the angle of inclination angle *α* is measured.

The reflective mirror M must ensure that the image of the laser spot on the measured surface is reflected to the original axis of the illumination laser beam to keep the sensor capability of distance measurement of the reflected laser spot image. In this case, the reflective plane of the mirror M must intersect the illumination laser beam at the angle *β*:

(5) $$\beta =\frac{90{}^{\circ}-\alpha }{2}.$$

If we assume the mirror M tip distance from the intersection point *l,* the total distance of the reflected mirror tip *L* from the intersection point with the illumination laser beam is given by the following relation:

(6) $$L=\frac{l}{\cos\beta }.$$

The range of the laser spot surface distances that can be measured along the reflected illumination laser beam is limited by two constraints. The minimum measurable surface distance equals the reflection mirror tip position in distance *l* in the direction of the reflected laser beam. The maximum distance is limited by the laser spot reflection by the reflection mirror M tip to the sensor’s lens aperture.

The modified sensor distance range can be evaluated using the corresponding values of the angle *φ* between the laser illumination beam and the laser spot imaging beam. As the reflected image of the laser spot at the closest distance *l* is reflected to the distance *B* + *l* along the original illumination laser beam, the corresponding angle *φ^M^_min_* can be computed from relation (3):

(7) $$\varphi_{min}^{M}=atan\left(\frac{A}{B+l}\right).$$

This angle gives a condition to the maximum mirror-free position *L_free_* from the laser beam intersection point in the mirror plane to allow reflection of the laser spot in distance *l* to the sensor lens aperture under the angle *φ*^M^_min_. For *L_free_*, the following is valid:

(8) $$L_{free}=l\frac{sin\varphi_{min}^{M}}{sin\left(\varphi_{min}^{M}+\beta \right)}.$$

The corresponding minimum reflection mirror M length *L_M_* to allow for the reflection of the laser spot within all ranges from *l* to *l_max_* distances is given by the following relation:

*L*_M_ = *L* − *L_free_*. (9)

The reflection mirror M tip angle *φ^M^_max_* can be calculated from the triangle given by the illumination laser beam reflection point, the reflection mirror M tip point, and the lens aperture point. The sensor viewing angle *ψ* covering the reflecting mirror M plane from angle *φ* to angle *φ^M^_max_* is given by the following relation:

(10) $$\psi ={\varphi -\varphi }_{max}^{M}=atan\frac{Lsin\chi }{C-Lcos\chi },$$

where *χ* is the angle between *C* and *L* distances, and it is equal to:

(11) $$\chi =\frac{\pi }{4}-\varphi +\alpha +\beta =\frac{3}{4}\pi -\varphi -\frac{\alpha }{2}.$$

The distance *E* in the original illumination laser beam direction corresponding to the distance *l_max_* in the reflected illumination beam direction can be computed with the help of relation (3), where the angle *φ* corresponds to *φ^M^_max_*. In this particular case, the distance E is given by the following relation:

(12) $$E=\frac{A-Btan\varphi_{max}^{M}}{tan\varphi_{max}^{M}}.$$

The maximum range *l_max_* of the laser spot surface distance measurement along the reflected illumination laser beam from the mirror M tip is given by the following relation:

*l_max_* = *E* − *l*. (13)

This maximum working range can also be expressed using the relation:

(14) $$l_{max}=L\frac{sin\beta }{tan\varphi_{max}^{M}}=Lsin\beta tan\left(\beta +\delta \right),$$

where *δ* is the angle between the principal ray reflected by the reflection mirror M tip and the reflective mirror M normal. It is given by the following relation:

(15) $$\delta =\beta +\alpha -\varphi_{max}^{M}.$$

The presented relations allow for the evaluation of any configurations of the commercial triangulation sensor modified with an attachment with a reflective mirror. Suppose that inclination angle *α* is smaller than the reflection mirror M tip angle *φ^M^_max_*. In that case, the imaging system lens position is right from the horizontal position of the reflective mirror M tip at distance *l*. It may lead to possible obscuration of the laser spot imaging beam by the measured vertical surface S. This obscuration can be minimized if the laser spot imaging beam is parallel with the vertical measured surface S. In this case, the position of the triangulation sensor imaging lens is placed in the same horizontal position above the reflective mirror M tip, as shown in Figure 2b. It corresponds to a PSA arrangement when the inclination angle *α* and the reflection mirror tip angle *φ^M^_max_* values are the same. In this case, the *δ* angle between the principal ray reflected by the reflection mirror M tip equals the mirror inclination angle *β* according to relation (15), where according to relation (5) becomes:

(16) $$\beta =\frac{90{}^{\circ}-\varphi_{max}^{M}}{2}.$$

In this case, the maximum working range given by relation (14) simplifies to:

(17) $$l_{max}=ltan\beta tan2\beta .$$

The presented equations provide ambiguous solutions of the attachment geometry under a selection of four independent input parameters. The selection of the triangulation sensor determines the distance *A* as the input parameter and the range of distance *B* or *φ* angle. The selection of other input parameters depends on the designer’s demands.

### 3.1. Design Based on Parameters A, l, and φ^M^_max_

The geometry of the attachment to the commercial triangulation sensor to allow side distance measurement can be determined based on four desired input parameters. The number of input parameters for the ambiguous solution of the attachment geometry reduces to three if we assume geometry that the laser spot imaging beam is parallel with the measured vertical surface S, leading to the condition:

(18) $$\alpha =\varphi_{max}^{M}.$$

Parameter *A*: The sensor’s imaging lens distance from the illumination laser beam is inherently given by the selected commercial triangulation sensor. It can be assumed to be the first fixed-value input parameter. The second input parameter can be considered as the future sensor probe diameter related to the horizontal distance l of the reflective mirror M tip from the illumination laser beam. The third input parameter can be the sensor inclination angle α value corresponding to the reflection mirror M tip angle *φ^M^_max_* according to relation (18).

In this case, the angle of reflection mirror inclination angle *β* is given by relation (16), and the corresponding measurement range is given by relation (17). The distance *E* in the original illumination laser beam direction corresponding to the desired maximum working range distance *l_max_* is given by the following relation:

(19) $$E=\frac{l}{\sin\varphi_{max}^{M}}.$$

The laser beam reflection mirror M has to be placed at distance *B* from the sensor’s imaging lens position in the direction of the illumination laser beam. This distance can be evaluated from relation (12):

(20) $$B=\frac{Acos\varphi_{max}^{M}-l}{sin\varphi_{max}^{M}}.$$

The total distance of the reflected mirror M tip from the intersection point with the illumination laser beam is given by relation (6). Finally, the minimum length of the reflected mirror LM to cover the whole working range from distance *l* to *l_max_* can be evaluated using the relations (7)–(9).

### 3.2. Design Based on Parameters A, l, and l_max_

Another important design constraint of the sensor system represents the demand for the desired maximum working range *l_max_*. As other input parameters can be assumed, the horizontal distance of the reflective mirror M tip from the illumination laser beam *l* and the selected commercial laser triangulation sensor gives the third input parameter value, the distance *A*.

As *l*_max_ and *l* distances are both known input values, the reflection mirror inclination angle *β* can be derived from relation (17):

(21) $$\beta =atan\sqrt{\frac{l_{max}}{2l+l_{max}}}.$$

The corresponding reflection mirror M tip angle *φ^M^_max_* equal to the sensor inclination angle *α* can be computed according to relation (16) as:

(22) $$\varphi_{max}^{M}=\alpha =90{}^{\circ}-2\beta .$$

Relations (20), (21) and (7)–(9) can be applied to reach the values of all other system design parameters.

### 3.3. Design of the Illumination Laser Beam Bypass

**Figure 3:**
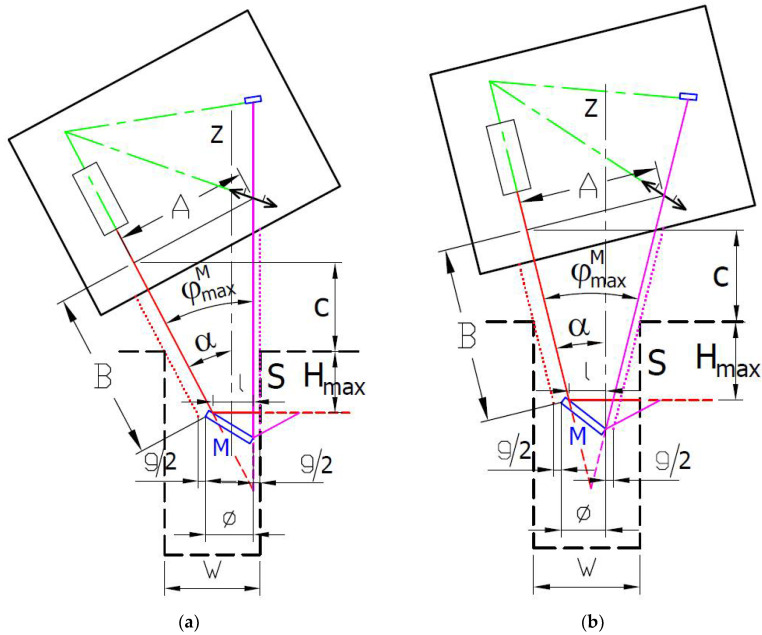
(**a**) The PSA arrangement scheme; (**b**) The SSA arrangement scheme.

The presented sensor modification designs correspond to the PSA arrangement, schematically shown in Figure 3a, where the inclination angle *α* equals the reflection mirror M tip angle *φ^M^_max_*. It assures that the laser spot imaging beam is obscuration-free. However, placing the reflection mirror holder into a narrow slot for distance measuring the surface S the inclination may lead to obscuration of the illumination laser beam of the sensor. It limits the depth of the side distance measurement for small bores and narrow slots. We can assess the aspect ratio of the measurement as the ratio of the maximum depth *H_max_* of side distance measurement to the bore or slot width *W* as:

(23) $$AR=\frac{H_{max}}{W}.$$

For the PSA arrangement, the measurement aspect ratio varies with the reflection mirror M tip angle *φ^M^_max_* of the particular sensor modification design and can be expressed by the following relation:

(24) $${AR}_{PSA}=\frac{H_{max}}{{\emptyset +g+H}_{max}tan\varphi_{max}^{M}},$$

where Ø = *l*+*e* is the footprint size of the mirror attachment, where the value *e* is given by the attachment design, and *g* is the safety gap between the attachment and the edge of the measured surface S. Reaching the values of *e* < 3 mm and *g* < 2 mm is feasible.

For the SSA arrangement, schematically shown in Figure 3b, the measurement aspect ratio can be expressed by the following relation:

(25) $${AR}_{SSA}=\frac{H_{max}}{{\emptyset +g+2H}_{max}tan\frac{\varphi_{max}^{M}}{2}}.$$

The SSA arrangement may reach a higher measurement aspect ratio because tan(*x*) > 2tan(*x*/2).

The issue with either laser illumination or imaging beam obscuration can be solved by redirecting the beams in the same or similar direction parallel to the measured surface S. It can be technically more easily performed in the case of PSA arrangement, where only one of the beams needs to be redirected. We proposed illumination beam redirection with bypass using three mirrors, M0, M1, and M2, as shown in Figure 2b. In this bypass arrangement (BA), the measurement aspect ratio can be expressed by the following relation:

(26) $${AR}_{BA}=\frac{H_{max}}{\emptyset +g}.$$

If we assume the BA design where the mirror M0 is placed close to the triangulation sensor housing reflecting the illumination laser beam in a horizontal direction, the inclination angle of the mirror M0 is the same as the angle *β* of the reflection mirror M given by relation (16).

The function of the mirrors M1 and M2 is to shift the illumination laser beam between two parallel directions the same as the periscope. In this case, its inclination angle *γ* needs to be the same and depend on the value of *ε* angle between the laser beam direction between mirrors M1 and M2 and the vertical axis *z* according to the following relation:

(27) $$\gamma =45{}^{\circ}-\frac{\varepsilon }{2}.$$

This result shows an important fact. The inclination angle of the illumination laser beam mirror M2 *γ* and the laser spot reflection mirror M angle *β* are not identical. A single mirror M can be used for both the reflection of the illumination laser beam and the laser spot reflection only if the incident illumination laser beam coincides with the sensor object plane. The illumination laser beam bypass changes the incident angle, and the sensor attachment has to be comprised of two differently inclined mirror surfaces.

### 3.4. Accessible Measurement Depth Evaluation

The presented bypass of the illumination laser beam path assures a small mirror attachment footprint of size Ø. The maximum accessible measurement depth *H_max_* of the sensor setup is proportional to the vertical distance between the periscopic mirrors M1 and M2. It is also proportional to the mirror M2 distance *B* according to the following relation:

(28) $$H_{max}\approx Bcos\varphi_{max}^{M}-c,$$

where *c* is a mirror attachment’s physical design-related input value limited by the sizes and positions of the mirrors M0 and M1 concerning the distance from the sensor case.

We have analyzed the geometrical possibilities of the presented sensor setup from the point of view of the accessible measurement depth *H_ma_*_x_ as a function of the mirror-attachment footprint proportional to distance *l* and the corresponding measurement working range *l_max_*.

If we assume the input parameters of the problem *A*, *l*, and *φ^M^_max_*, the accessible measurement depth *H_max_* can be evaluated using relations (24) and (20), resulting in the following relation:

(29) $$H_{max}\approx \frac{Acos\varphi_{max}^{M}-l}{tan\varphi_{max}^{M}}-c.$$

The corresponding maximum working range is given by relation (14).

**Figure 4:**
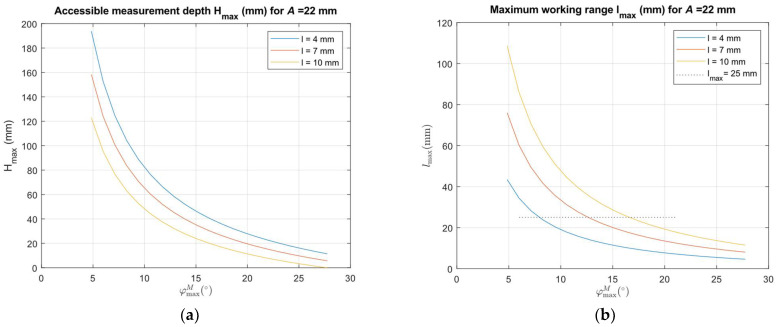
(**a**) Values of accessible measurement depth *H_max_* as a function of the sensor inclination angle *φ^M^_max_* = *α* for selected values of the reflection mirror tip distance *l*; (**b**) Values of accessible maximum working range *l_max_* as a function of the sensor inclination angle *φ^M^_max_* = *α* for selected values of the reflection mirror tip distance *l*. The dotted horizontal line indicates *l_max_* = 25 mm.

Figure 4 shows the resulting values of accessible measurement depth and maximum working range as a function of the reflection mirror M tip angle *φ^M^_max_* for selected values of the reflection mirror M tip distance *l* and *A* = 22 mm and *c* = 18 mm.

**Figure 5:**
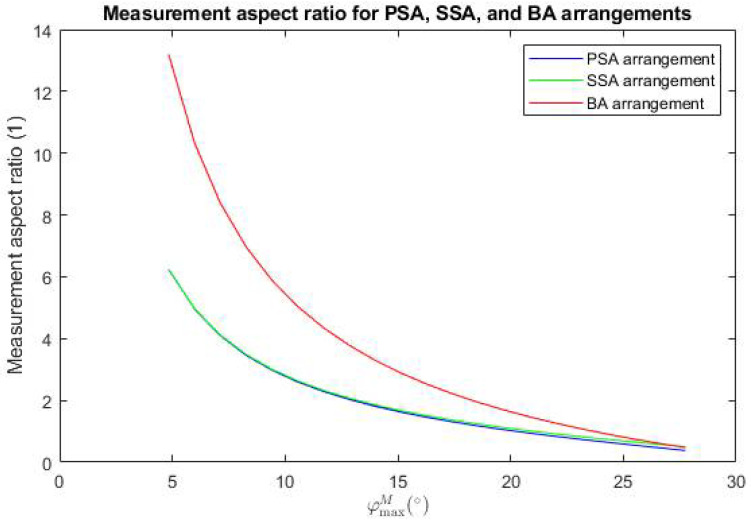
The measurement aspect ratio for SSA, PSA, and BA arrangements as a function of the reflection mirror M tip angle *φ^M^_max_*, for *l* = 7 mm, *e* = 2 mm, *g* = 2 mm, *A* = 22 mm, and *c* = 18 mm.

We also compared the measurement aspect ratio as a function of the reflection mirror M tip angle *φ^M^_max_* for SSA, PSA, and BA arrangements for values *l* = 7 mm, *e* = 2 mm, *g* = 2 mm, *A* = 22 mm, and *c* = 18 mm in Figure 5.

Figure 5 shows that the SSA arrangement can reach a measurement aspect ratio higher than the PSA arrangement, especially for high *φ^M^_max_* angles. However, with decreases in the *φ^M^_max_* angle (for sensors with longer working range), the BA arrangement can reach a higher measurement aspect ratio than any of the arrangements using a single reflective mirror.

### 3.5. Influence of the Imaging Beam Size

**Figure 6:**
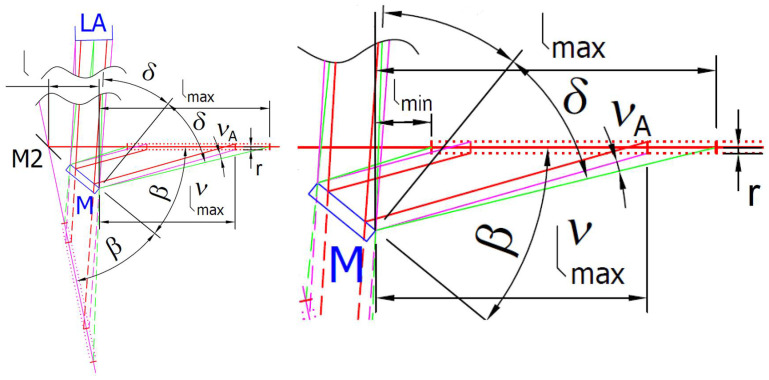
Scheme of the sensor working range reduction caused by real sizes of the illumination laser beam, and the lens aperture. The real laser beam size is indicated with a red dotted line. The principal rays are green. The rays reflected from the laser spot rim to the lens aperture rim by the mirror edge are magenta. The rays reflected from the laser spot rim by the mirror M surface are red.

All presented relations are derived for the principal rays among the laser spot center, the center of the imaging lens aperture, and the reflective mirror M tip position. It neglects the real size of the laser spot and imaging beam reflecting on the mirror edge and entering the real lens aperture size. The principal ray analysis describes the situation in which only one-half of the laser spot is reflected by the mirror edge, as schematically shown in Figure 6. It leads to the redistribution of the laser spot image intensity profile, which affects the commercial sensor distance processing algorithm.

If we assume the laser spot radius *r* and the lens aperture LA viewing half-angle *ν_A_*, the laser spot is fully reflected by the mirror M surface up to distance *l^ν^_max_*. It is given by the following relation:

(30) $$l_{max}^{\nu }=tg\left(2\delta -\nu_{A}\right)\left(ltg\beta -r\right),$$

where the angle *δ* is equal to the angle *β* for *φ^M^_max_* = *α*.

Similarly, if the mirror M length is smaller than the *L_M_* value given by relation (9), the modified sensor working range start is moved to the distance *l_min_* for the principal ray. According to relation (8), it gives the following relation:

(31) $$l_{min}=L_{freeM}\frac{sin\left(\varphi_{min}^{M}+\beta \right)}{sin\varphi_{min}^{M}}-l_{M},$$

where *L_freeM_* is the mirror-free distance to the laser beam intersection point and *l_M_* is the real mirror length *L_M_* projected to the reflected illumination laser beam, given by relation (6). If we assume full laser spot size reflection on the mirror M, it shortens the modified sensor working range to start at the distance *l^ν^_min_*, given by the following relation:

(32) $$l_{min}^{\nu }=tg(2\delta_{min}+\nu_{A}+\varphi_{min}^{M}-\alpha )((l-l_{M})tg\beta +r)-l_{M}.$$

Our previous measurement [25] showed that a small fraction of the imaging beam obscuration does not affect the sensor distance processing algorithm implemented by the sensor producer. Therefore, distances *l^ν^_min_* and *l^ν^_max_* represent the worst estimate of the correct sensor distance measurement range without mirror edge reflections affecting the sensor processing algorithm.

## 4. Experimental Probe Design

To prove the functionality and measurement possibilities of vertical surfaces of slots and bores, we designed a probe system composed of a common commercial triangulation sensor and the mirror attachment.

The typical aim for the design of the sensor mirror attachment is the need for distance measurement in a limited space smaller than the common distance sensor’s external dimensions. If we assume the sensor sizes and distance of the start of the sensor measurement range, the side distance measurement of the bore or slot of width less than 50 mm can be an issue. It raises a typical demand for the sensor attachment design to input the maximum distance range value *l_max_*. The second typical constraint of the attachment design is the available laser triangulation sensor. It limits design freedom with the sensor working range from *d_min_* to *d_max_* and corresponding *A* and *B* distances. Sensor distance *A* can be found in the sensor datasheet or estimated using the sensor geometry measurement. We assume *A* = 22 mm for the available Micro-Epsilon Messtechnik GmbH & Co. KG (Ortenburg/Germany) optoNCDT ILD 1420-200 laser displacement sensor (1D). The third design input condition can be the distance *l* of the reflective mirror M tip or the condition of the sensor inclination expressed by angles *φ^M^_max_* and/or *α*. We decided to perform the sensor mirror attachment design based on parameters *A*, *l*, and *l_max_* according to Section 3.1. The sensor’s maximum working range for the principal rays *l_max_* = 25 mm was assumed as the desired value.

The desired *l_max_* value can be achieved using various sensor designs, as shown in Figure 4b with a dotted line. The corresponding design parameters for the selected mirror M tip distance *l* and *l_max_* = 25 mm are evaluated from the presented equations and summarized in Table 1.

The selection of a particular design is limited by values of distances *B* + *l* and *B* + *E*, which represent the minimum and maximum distances that need to be covered by the sensor’s working range. Table 1 shows the highest aspect ratio of accessible depth *H_max_* to mirror footprint size Ø > *l*, which can be achieved using a small mirror M tip distance *l* and sensors with a long working range (*B* + *E* distance). On the other hand, such a design can be affected by issues caused by the stiffness of a long mirror holder and high demands for precise alignment of small mirrors to the desired position where the reflective surface of the mirror M2 has to be placed into an intersection of the sensor’s original illumination laser beam axis, the bypassed laser beam axis, and the reflective mirror M surface plane. For this reason, we decided to realize the sensor attachment geometry with the laser spot reflection mirror M tip distance *l* = 7 mm and the laser beam reflection mirror M2 placed at the distance *B* = 66.14 mm. As a sensor, we used the Micro-Epsilon optoNCDT ILD 1420-200 laser displacement sensor, which allows a minimum measurement distance of 60 mm and a maximum distance of 260 mm, respectively. The linearity of this sensor was ≤0.08% of the full scale, corresponding to 160 μm. The repeatability of the ILD 1420-200 sensor was 8 μm. The laser spot size is 750 × 1100 μm at the start of the measurement range and diverges along to the end of the measurement range.

**Figure 7:**
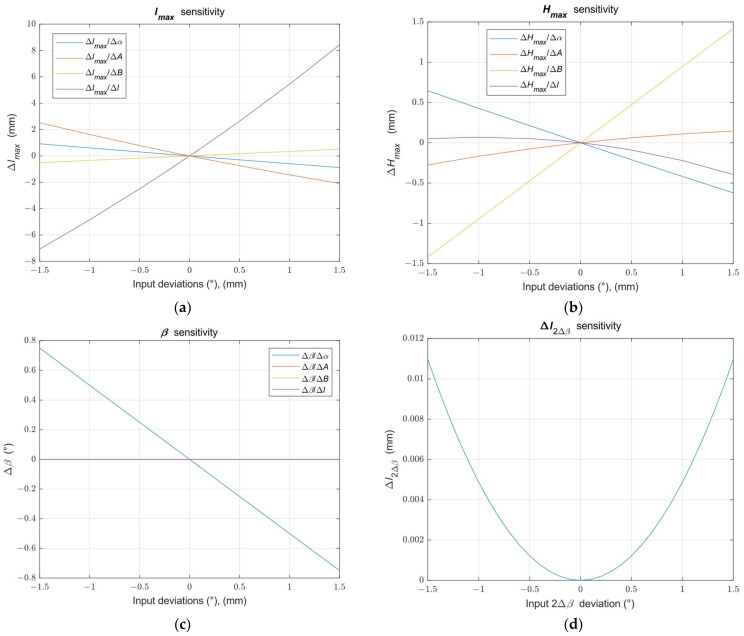
Sensitivity of the target parameters to the variations of the input parameters the distances *A*, *B*, and *l*, and angle *α*. (**a**) The sensitivity curves of the maximum working range *l*_max_; (**b**) The sensitivity curves of the maximum measurement depth *H_max_*; (**c**) The sensitivity curves of the reflection mirror M inclination angle *β*; (**d**) The sensitivity curves of the measured distance error Δ*l*_2Δβ_ on an error angle 2Δ*β* of the reflected illumination laser beam.

The distances and angles of the sensor system provided with the attachment may differ from the values assumed in Table 1 in physical realization. For this reason, we evaluated the sensitivity of the target parameters to variations in the input parameters. We present the sensitivity curves in Figure 7.

One source of input data uncertainty is the real values of distances *A* and *B*. If the sensor manufacturer does not provide the necessary information, the position of the sensor imaging lens aperture relative to the illumination laser beam and the sensor housing must be estimated using sensor geometry measurements. In this case, the values of distances *A* and *B* can be affected with a high uncertainty of up to ±1.5 mm. Another source of uncertainty is the real values of the mirror M tip distance *l* and the inclination angle *α* of the illumination laser beam set during the assembly of the system. The graphs in Figure 7a,b represent the influence of the uncertainty of all input parameters on the target values of the maximum working range distance and the maximum measurement depth.

The maximum working range distance *l_max_* appears as an almost linear sensitivity of all input variables in the range ± 1.5 mm of distance deviations and ±1.5° variation of the inclination angle *α*. The maximum working range *l_max_* is the most sensitive in the change of the mirror M tip distance *l*. The system design must ensure high accuracy in the *l* distance setting to achieve an acceptable uncertainty of the distance *l_max_* in the range ± 1 mm.

The maximum working depth *H_max_* usually shows a non-linear sensitivity to changes in input parameter values but is less sensitive than the maximum working range distance.

The influence of every input parameter uncertainty on the mirror angle *β* and their effect on the measured distance error is shown in the graphs in Figure 7c,d. It shows that the mirror angle *β* depends only on the inclination angle *α* error, where the relation between the error angles is Δ*β* = Δ*α*/2.

(33) Δβ=Δα/2.

However, since the illumination laser beam is reflected from the mirror to the double angle, the total deviation of the reflected laser beam is 2Δ*β*. Changing the mirror angle *β* causes the reflected illumination laser beam to be not directed perpendicular to the measured surface but at an error angle 2Δ*β*, and the measured surface distance *l_S_* from the mirror M tip is affected by the cosine error given by the following relation:

(34) $$\Delta l_{2\Delta \beta }=\left(l+l_{S}\right)\left(1-cos2\Delta \beta \right).$$

In our design case, the deviation 2Δ*β* = 1.2° corresponds to the repeatability of the distance measurement declared by the sensor manufacturer. This increases the requirement to set the angles of the sensor with the attachment with an uncertainty of better than 0.5°. On the other hand, the relation (33) between the whole setup inclination angle error allows the compensation of the reflected laser beam by rotating the installation angle of the whole setup in the opposite direction. This would require placing the measurement system on a fixture, allowing this rotation adjustment possibility.

In addition to the specified requirements for the accuracy of distances and angles of the measurement system, the setup assembly must meet condition (b): the mirror reflection surface has to be symmetrically placed between the original laser beam direction and the reflected laser beam direction. This condition is inherently satisfied by an attachment using a single mirror that reflects both the illumination and imaging beams. The improper assembly of a BA arrangement composed of separated mirror surfaces to reflect the illumination and imaging beams may violate this condition. If the laser spot on the measured surface is imaged outside the depth of field of the sensor imaging optics corresponding to the size of the illumination laser beam, an error in the measured distance occurs due to a change in the slope of the sensor linearity. For this reason, we have developed an assembly procedure to eliminate the mentioned errors.

The sensor probe attachment consists of two parts to allow its mutual adjustment. The first part was a 3D-printed part acting as a sensor holder in the appropriate position with respect to the external coordination system used for the probe scanning motions. It also carries two bypass mirrors, M0 and M1. The same part is also used to fix a second part of the attachment, carrying the laser beam reflection mirror M2 and the laser spot reflection mirror M for the vertical surface distance measurement. The carrier part with mirrors M2 and M was fixed to the sensor holder part with a clamp joint to allow for the mirror vertical position adjustment. For the illumination laser beam bypass mirrors M0 and M1, we used Edmund Optics 6 × 6 mm Enhanced Aluminum, 4–6λ Mirror. For the illumination laser beam reflection mirror M2, we used Edmund Optics 3 × 3 mm Enhanced Aluminum, 4–6λ Mirror. For the laser spot reflecting mirror M, we used the mirror Edmund Optics 4–6 wave 6.3 mm with the possibility to ground the mirror size to the desired mirror length. We used this possibility to test the influence of the shortened mirror’s edge on the sensor modification linearity measurement. In that case, the mirror was ground to a length *L_M_* = 5.75 mm. The total size of the sensor attachment footprint in the position of mirrors M2 and M was Ø = 7.8 mm. The sensor attachment footprint spreads towards its fixing, reaching Ø = 11.6 mm due to bypassed illumination laser beam access. It allows the safe insertion of the whole length of the sensor’s attachment inside a hole more than 12 mm in diameter.

The mirror carrier and the sensor holder parts were 3D printed with the SLS technique on a PRUSA SL1S 3D printer (Prague, Czech Republic). We used Prusa Resign—Tough (Gray and Black) resins (strength limit 52 MPa, Young modulus 1.25 GPa) with a 0.05 mm layer to ensure good stiffness, surface quality, and fine precision for setting the mirror position.

The sensor attachment setup assembly was performed using a stereomicroscope and the Nikon iNEXIV VMA-2520 (Tokyo, Japan) automated measuring system. We also used specially designed assembly tools to hold individual parts and to indicate the laser beam direction setting. The assembly uncertainty was limited by the determination of the position and angle of the illumination laser beam axis. Using the mentioned instruments and tools, the uncertainty of the set dimensions can be achieved to ±0.1 mm and the set of angles to ±0.2°.

The first step of the system assembly was to fix the mirrors M and M2 to the carrier part at the correct angle and correct distance *l* of the tip of the mirror M from the intersection point of their reflecting surfaces. This intersection point was marked as a reference for further adjustment of the carrier part relative to the sensor holder part. The sensor was fixed to the sensor holder part using an assembly tool to indicate the setting of the angle *α* to the vertical axis Z using the sensor illumination laser beam. The axis of the illumination laser beam was used as a reference for the next adjustment of the marked intersection of mirrors M and M2 to the correct position *B* and angle *β* using the direction of the illumination laser beam after reflection on mirror M2. After reaching the desired position, the carrier part with mirrors M2 and M was fixed to the sensor holder part with a clamp joint. In the final stage, the sensor holder part was fitted with mirrors M0 and M1 to redirect the illumination laser beam and mirror M1 was adjusted to the reflected beam to meet the marked intersection of mirrors M2 and M. Incorrect assembly of the entire setup was indicated by measuring the distance when the deviation of the data slope was higher than linearity tolerance declared by the sensor manufacturer. In the correct assembly, the deviation of the reflected illumination laser beam from a direction perpendicular to the *Z*-axis was less than 0.5°. It was unnecessary to compensate for this by adjusting the whole setup inclination angle *α*.

**Figure 8:**
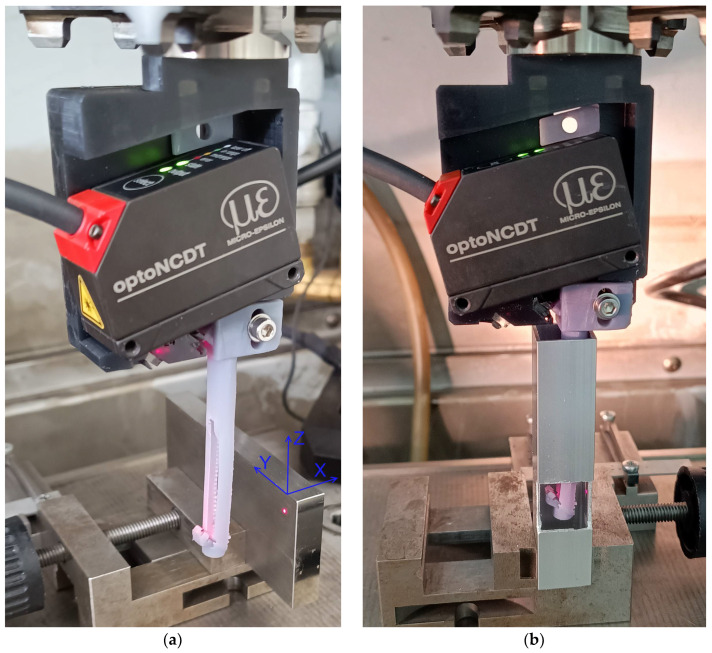
The sensor system with double mirrors M2 and M attachment during measurement: (**a**) A probe linearity measurement using the vertical surface in distance X = −25 mm, Z = −5 mm represented by the ground steel block illuminated with the laser spot; (**b**) Demonstration of the high aspect ratio (3.5) in-depth distance measurement inside the 13 mm wide slot in-depth Z = −46 mm below the upper edge of the measured surface.

The whole probe was provided with an EROWA ITS Chucking spigot (Büron, Switzerland) to allow its fixing to the machine chuck. We used the four-axis EDM Sodick AP1L machine (Schaumburg, IL, USA) to achieve the precise scanning motion of the system. The machine provides translation for X, Y, and Z with uncertainty ±2 µm and vertical rotation axis indexation. Figure 8 shows the image of the modified sensor system under test conditions.

## 5. Results

We aimed to test the probe for the measurement of the vertical surface distance demonstrated with a gauge block fixed to the machine magnetic table. The first step was to define the offsets between the machine coordination system and the real mirror M tip position. We set the mirror edge close to the block side edge and checked its position with 0.4X SilverTL™ Telecentric Lens (Edmund Optics, Barrington, NJ, USA) and DMKUX178 monochrome 6MP Imaging Source camera (The Imaging Source, Charlotte, NC, USA) in the *X*- and *Z*-axis directions with the block edge. For safety reasons, the machine coordination system did not coincide with the block edge, but the safety gap was set between the mirror edge and the measured surface. This gap may vary when the machine is turned off; thus, we checked the real probe position before every measurement.

### Probe Linearity Measurement

**Figure 9:**
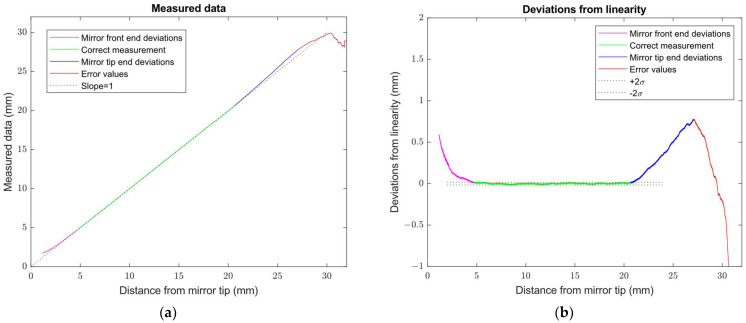
Measurement of the linearity of the sensor probe in the horizontal *X*-axis motion: (**a**) Sensor distance data as a function of the real distance from the reflective mirror M tip; (**b**) Deviations from the linear approximation of the sensor correct distance measurement range. The dotted lines represent the linearity slope and repeatability value guaranteed by the manufacturer of the commercial sensor used.

The linearity measurement of the sensor probe consisted of the measurement of the distance in the direction of the *X*-axis perpendicular to the vertical surface of the block, shown in Figure 8a. This kind of probe motion ensured that the laser spot was incident on the fixed part of the block’s surface all the time and minimized the influence of the block’s surface roughness. The surface distance was measured with a constant velocity motion of 0.4 mm/s starting at X = 0 of the position of the machine coordination system. The reflective mirror M tip distance from the measured surface was 2.165 mm for X = 0. The linearity measurement was performed in five Z positions of the laser spot below the upper edge of the block (Z = 0), corresponding to the Z-values of the machine coordination system [−5, −10, −15, −20, −40]. Typical measured data are shown in Figure 9.

The sensor provided distance data from the start position of the measurement X = 0 up to a distance of about 32 mm from the mirror M tip, where an error status was indicated. The measured values are shown in Figure 8a. Error values are marked as a 1 mm distance step from the last sensor value. Figure 8b shows deviations from the linear approximation of the measured data. In addition to the correct measurement range indicated in green, there are visible distances with high deviations from the linear characteristics caused by the laser spot reflection close to the mirror M edges, as shown in Figure 4. Short distances indicated in magenta are affected due to the use of a shorter mirror length LM than the minimum reflection mirror length given by relation (9) and evaluated in Table 1. In this case, the distance *l_min_* = 2.414 mm according to Equation (27), under the realized design *l* = 7 mm and the mirror M length *L_M_* = 5.75 mm. At the *l_min_* distance, the principal ray crosses the illumination laser axis. If we assume that the sensor lens aperture diameter is 5 mm and the radius of the illumination laser beam *r* = 0.4 mm, the sensor working range starts at distance *l^ν^_min_* = 4.814 mm according to Equation (28). At *l^ν^_min_* distance, the laser spot rim is reflected to the lens aperture rim by the mirror M edge. It represents the worst estimate of the correct sensor distance measurement range start as the sensor processing algorithm is not sensitive to partial laser spot beam obscuration.

Long distances are affected by the mirror M tip edge reflection. A design distance *l_max_* = 25 mm is valid only for the principal ray. If we assume that the laser spot radius expanded to radius *r* = 0.5 mm and the lens aperture diameter, the end of the sensor distance measurement range corresponds to the distance *l*^ν^*_max_* = 19.288 mm for the laser spot rim reflected to the lens aperture rim by the mirror M edge. It also represents the worst estimate of the end of the correct sensor distance measurement range.

We processed measured data for evaluation distances corresponding to the start *l^ν^_min_* and end *l*^ν^*_max_* of the sensor’s correct measurement range. We also evaluated the positions of the measured distances maximum error *l_max dev_* and the error indication *l_err_* position. The results are summarized in Table 2 for the individual measurement position Z.

The results show a relatively high variability of the monitored distances with a mean standard deviation of 0.275 mm. On the other hand, the average value of the start *l^ν^_min_* = 4.355 mm of the sensor’s correct measurement range is correctly lower than the worst estimate given by Equation (28) for the distance *l^ν^_min_* = 4.814 mm. It corresponds to the sensor’s laser spot processing algorithm, allowing for about 15% obscuration laser spot beam without affecting the distance values. Similarly, the average value of the end *l^ν^_max_* = 20.44 mm of the sensor’s correct measurement range is correctly greater than the worst estimate given by Equation (26) distance *l^ν^_min_* = 19.288 mm. It extends the usable range of correct distance measurement above the distance of the worst estimate valid for the rays reflected from the laser spot rim to the lens aperture rim.

**Figure 10:**
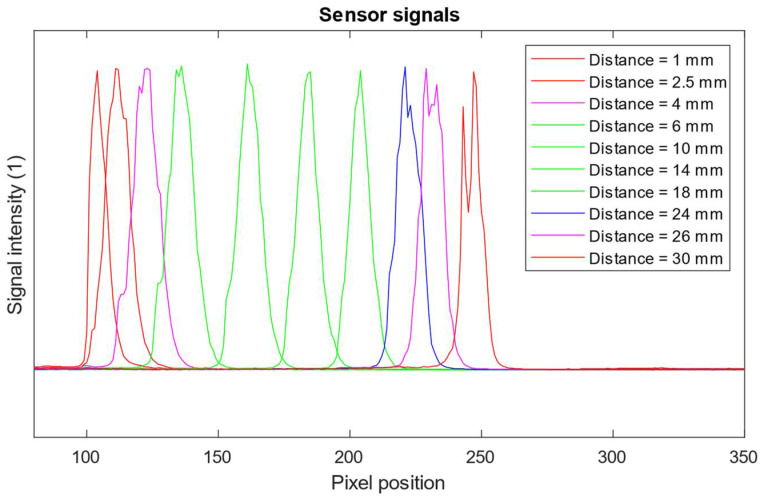
The laser spot image profiles gathered by the sensor’s linear detector; correct distance data—green, deviated distance data—magenta and blue; error signal positions data—red.

The modified sensor provides distance data even for distances greater than *l^ν^_max_*, but the indicated values deviate from the real distance. We evaluated the change in the slope of the deviated distance data indicated in blue in Figure 9 compared to the correct distance measurement range in green. The average slope changes from 1 for the correct measurement range to about 1.126 for the deviated distance data range. It causes the magnification of any change in distance compared to the real distance. When the laser spot position leaves the possibility of being reflected by the reflective mirror M to the lens aperture, the sensor indicates an error status. The laser spot image profiles corresponding to the correct distance data and the deviated distance data due to the obscuration of the laser spot imaging beam are shown in Figure 10.

All laser spot image profiles shown in Figure 10 were processed by the sensor laser spot processing algorithm. However, since subpixel peak extraction algorithms typically assess a limited number of profile data points [28], redistribution of the intensity profile due to the obscuration of the laser spot beam leads to incorrect distance value.

**Figure 11:**
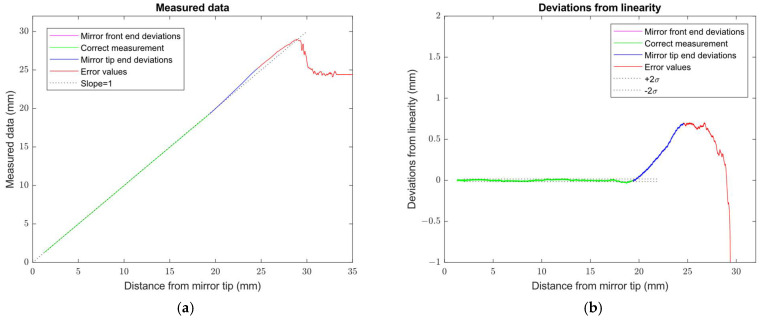
Sensor probe with real mirror length *L_M_* = 6.4 mm: (**a**) Sensor distance data as a function of the real distance from the reflective mirror M tip; (**b**) Deviations from the linear approximation of the sensor correct distance measurement range. The dotted lines represent the linearity slope and repeatability value guaranteed by the manufacturer of the commercial sensor used.

When we removed a shortened ground reflective mirror by a full-length mirror *L_M_* = 6.4 mm, the modified sensor characteristics extended the correct measurement range to distances close to the mirror M tip, as shown in Figure 11.

## 6. Discussion

We proposed and demonstrated the possibility of modifying a commercial laser triangulation sensor to measure the vertical surface distance of high aspect ratio slots or bores at a depth corresponding to multiples of its size. We have derived the necessary relations for a commercial sensor mirror attachment design. It allows one to evaluate the side distance measurement range at the desired depth based on the sensor parameters and the size and position of the reflective mirror. We also solved the inverse problem of achieving the mirror attachment geometry based on the desired side distance measurement range at the desired depth. We analyzed the sensitivity of the modified sensor geometry to variations of the four input parameters, solving the presented relations. We have detailed the assembly procedure to maintain the uncertainty limits of used commercial sensors.

We also analyzed the influence of the real size of the laser spot and the lens aperture on the side distance measurement range. The effect of the laser spot obscuration by the reflective mirror edges shortens the range of correct side distance measurement compared to relations using propagation of the principal rays. We proposed relations to the worst estimate of the correct sensor distance measurement range. If we admit that the sensor distance processing algorithm tolerates the obscuration of a small fraction of the laser spot imaging beam, the real correct distance measurement range exceeds the proposed worst estimate.

**Figure 12:**
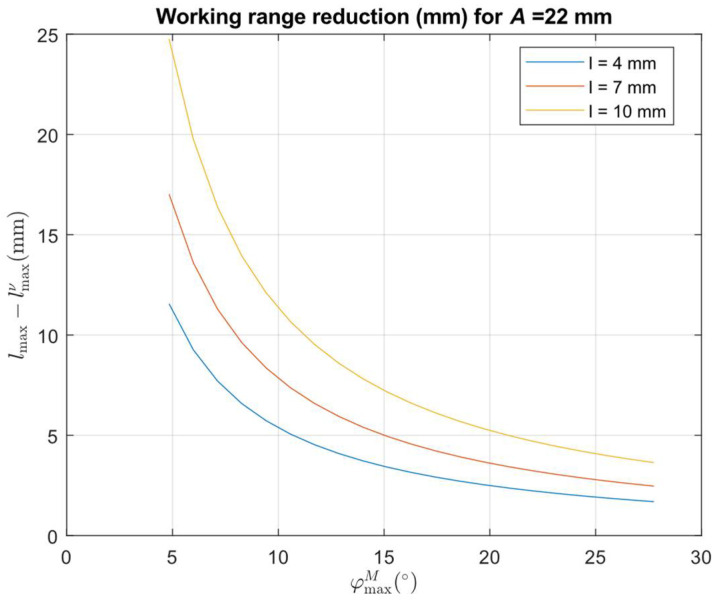
Values of the reduction of the maximum working range *l_max_* to the minimum distance assumption of the end of the linear part of the working range as a function of the sensor inclination angle *φ^M^_max_* = *α* for the selected values of the reflection mirror tip distance *l*.

The theoretical analysis presented in Figure 4 shows the possibility of designing a sensor attachment that can perform the side distance measurement in depth up to 16 times the sensor attachment footprint size by placing a mirror tip at a distance of 4 mm from the illumination laser beam at a distance of 129 mm. It requires a sensor with a maximum working position of at least 160 mm. It will provide distance data up to the measured surface distance from the mirror M tip 42 mm. But, due to the real size of the laser beam, the maximum non-affected distance value will be reduced to 30 mm. Figure 12 presents differences between the *l_max_* values, which are valid for the principal rays and the minimum distance assumption of the end of the linear part of the working range caused by the real size of the illumination beam keeping the uncertainty limits of the used commercial sensor.

It allows the user to simply increase the target value of *l_max_* to reach a desired usable sensor working range of the used commercial sensor while keeping its uncertainty limits.

The graphs presented in Figure 4 and Figure 12 show that the attachment enables distance measurement in critical bore or slot sizes below 50 mm using relatively small mirrors with the tip placed at a distance *l* below 10 mm. Smaller distances *l* provide deeper depth access but reduce the maximum side distance range. Using larger mirrors results in an increase in the maximum side distance range but is not effective since bores or slots larger than 50 mm can be measured by a small housing-size triangulation sensor without the need for mirror attachment. The charts also show that the same maximum side distance range can be achieved with the reflective mirror placed at different distances from the sensor housing. It allows us to adapt the attachment design to the currently available triangulation sensor.

For measuring side distances in a slot or bores with a high aspect ratio, a narrow attachment profile must be maintained along the whole distance from the reflection mirror to the sensor housing. The distance between the illumination laser and the sensor imaging axes usually overcomes the small mirror-size attachment footprint. This would reduce the maximum accessible measurement depth. We solved this issue by bypassing the laser illumination beam using three additional mirrors.

We demonstrated the functionality of the sensor modification with an attachment employing the illumination laser beam bypass mirrors capable of reaching side distance measurement in depth up to 47 mm. We aimed to test the modified sensor linearity limits for the side distance measurement range, which was predicted as the worst assumption regarding the laser spot size and the sensor optics aperture size. The results show the correct prediction of the real correct measurement range. The measured side distance measurement range was greater than the worst-case predicted value due to the ability of the sensor distance processing algorithm to process even a partially obscured laser spot beam. The modified sensor with the proposed attachment performed a side distance measurement at the predicted depth. We have demonstrated the ability to perform side surface distance measurements inside a 13 mm width slot. The measurement aspect ratio allowed for an in-depth measurement corresponding to more than 3.5 times the slot width.

We hope that the proposed relations and the sensor attachment design approaches will help the sensor users extend the laser triangulation sensor distance measurement capability for a new kind of surface geometry.

## Figures and Tables

**Table 1:** Sensor mirror attachment design parameters for selected *l_max_* = 25 mm and *l* distances.

Parameters	Units	*l* = 4 mm	*l* = 7 mm	*l* = 10 mm
*l_max_*	(mm)	25	25	25
*A*	(mm)	22	22	22
*α*	(°)	7.928	12.636	16.602
*β*	(°)	41.036	38.682	36.699
*φ^M^_max_*	(°)	7.928	12.636	16.602
*φ^M^_min_*	(°)	9.394	16.742	24.271
*E*	(mm)	29.000	32.000	35.000
*B*	(mm)	128.975	66.136	38.790
*B* + *l*	(mm)	132.975	73.136	48.790
*B* + *E*	(mm)	157.975	98.136	73.790
*L_free_*	(mm)	0.847	2.449	4.701
*L_M_*	(mm)	4.456	6.518	7.771
*H_max_*	(mm)	109.743	46.754	19.173

**Table 2:** Summary of measured sensor distance measurement range limits.

(mm)	Z = −5	Z = −10	Z = −15	Z = −20	Z = −40	Average	Std	Expected
*l* ^ν^ *_min_*	4.389	4.709	4.317	3.932	4.429	4.355	0.279	<4.814
*l* ^ν^ *_max_*	20.605	20.277	20.553	20.468	20.298	20.440	0.148	>19.288
*l* *_max dev_*	26.781	27.069	26.853	26.596	25.797	26.571	0.473	~25
*l* *_err_*	31.981	32.525	32.110	32.276	32.469	32.272	0.231	-
Slope Dev	1.9093	1.131	1.128	1.137	1.143	1.126	0.020	-

## Data Availability

The original contributions presented in the study are included in the article, further inquiries can be directed to the corresponding author.
